# Early-Onset Schizophrenia in a paediatric population of French psychiatric and medico-social care centres: A cross sectional study

**DOI:** 10.1371/journal.pone.0236241

**Published:** 2020-07-27

**Authors:** Emmanuelle Dor-Nedonsel, Marie-Line Menard, Arnaud Fernandez, Charlotte Sakarovitch, Eric Fontas, Xavier Salle-Collemiche, François Poinso, Anne-Lise Tosello, Fanny Maria, Valeria Manera, Florence Askenazy, Susanne Thümmler

**Affiliations:** 1 University Department of Child and Adolescent Psychiatry, Children’s Hospitals of Nice CHU-Lenval, Nice, France; 2 CoBTek, University Côte d’Azur, Nice, France; 3 Direction de la Recherche Clinique, University Hospital, Nice, France; 4 University Department of Child and Adolescent Psychiatry, Public Assistance- Hospitals of Marseille, France; University of Toronto, CANADA

## Abstract

**Background:**

Early-Onset Schizophrenia (EOS) is rare but severe mental health disorder in children and adolescents. Diagnosis of schizophrenia before the age of 18 years remains complex and challenging, especially in young children. In France, there are no recent reliable epidemiological data about the prevalence of EOS. The present study evaluates the EOS rate in a target clinical population of children and adolescents in psychiatric and medico-social care centres in the South-East of France.

**Methods:**

Psychiatric and medico-social centres for children and adolescent in the geographical area have been contacted, and after receiving their agreement to participate in the study, eligible patients corresponding to inclusion criteria were selected based on patients’ medical records. Main inclusion criteria were age 7 to 17 years and intelligence quotient > 35. EOS categorical diagnosis was assessed by Kiddie-SADS Present and Lifetime psychosis section.

**Results:**

37 centres participated and 302 subjects have been included in the study. The main result was the categorical diagnosis of EOS in 27 subjects, corresponding to a rate of 8.9% in the study population. Half of the patients presented mild to moderate intellectual deficiency. Interestingly, only 2.3% had a diagnosis of schizophrenia spectrum disorder noted in their medical records before standardized assessment.

**Conclusions:**

The results of the study highlight the importance of using a standardized diagnostic tool for the diagnosis of schizophrenia in the paediatric population. In fact, EOS might be underdiagnosed in children and adolescents with neurodevelopmental disorders and subnormal cognitive functioning.

**Trial registration:**

NCT01512641. Registered 19 January 2012; https://clinicaltrials.gov/ct2/show/NCT01512641

## Background

Diagnosing schizophrenia before 18 years remains very complex. Indeed, there are no specific criteria for Early-Onset Schizophrenia (EOS) in standard classifications such as ICD-10 [1], DSM IV-TR [2] or DSM-5 [3]. To date, the prevalence of EOS has not been clearly identified. There is only a very limited number of epidemiological studies. Incidence data with diagnoses based on standardised clinical assessments such as the Schedule for Affective Disorders and Schizophrenia for School Age Children (Kiddie-SADS-PL) [4], are still rare in the paediatric population [5, 6]. Epidemiological studies concerning EOS are rare and of heterogeneous results [7, 8]. Observations from the National Institute of Mental Health cohort indicate that Childhood-Onset Schizophrenia (COS), with onset before 13 years, is very rare with an incidence of less than 0.04% [7]. In another study, prevalence is described as less than one in 10,000 children between 2 and 12 years of age [8]. In addition, other studies revealed that EOS represents less than 4% of all the schizophrenia diagnoses [6]. Specific and general care for EOS remains difficult and very challenging, and its outcome is worse than for other psychotic disorders [9].

In France, children and adolescents with neurodevelopmental disorders also including EOS are likely to be treated in the medico-social sector (MSS) offering educational support and the child psychiatric health sector (PHS) focusing on psychiatric care. Various types of institutions funded by Social Security (government subsidies), have been developed in both areas accommodated to the specific needs of the populations.

The French Equality of Rights and Chances law ensures that disabled persons in each sub-region [10] are directed to adapted MSS structures, based on detailed criteria including clinical, psychosocial and intellectual ability assessment. The institutions are composed of multidisciplinary teams, with psychiatrists being present only a few hours per week.

Orientation towards MSS or PHS is based on the clinical judgment of the referring psychiatrist according to ICD-10 [1], without mandatory standard diagnostic assessment. In consequences, there is no real benchmark of EOS rate in France.

According to our clinical experience, we hypothesised that EOS is underdiagnosed in current practice in France among the population of children and adolescents integrated in the centres described above.

The aim of this study was to estimate the rate of EOS in the pædiatric population integrated in medico-social centres or in psychiatric outpatient care in South-East France using a standardized instrument, the Kiddie-SADS-PL [4]. In addition, the study aimed at characterising the clinical and neurocognitive profile of patients diagnosed with EOS.

## Methods

The study has been approved by the Ethic Committee “Sud Méditarranée V” (ref.2011-A00787-34) and the French National Agency for Medicines and Health Products Safety (ANSM, n°B111395-70). The study has been registered on ClinicalTrials.gov (NCT01512641).

### Design of the study

The study aimed at exploring the rate of the categorical EOS diagnosis using the psychosis section of the Kiddie-SADS-PL [4] and was part of a broader study investigating dimensional EOS diagnosis and neurocognitive exploration of subjects fulfilling DSM-IV-TR diagnostic criteria for schizophrenia.

### Selection of centres (Fig 1)

**Fig 1:**
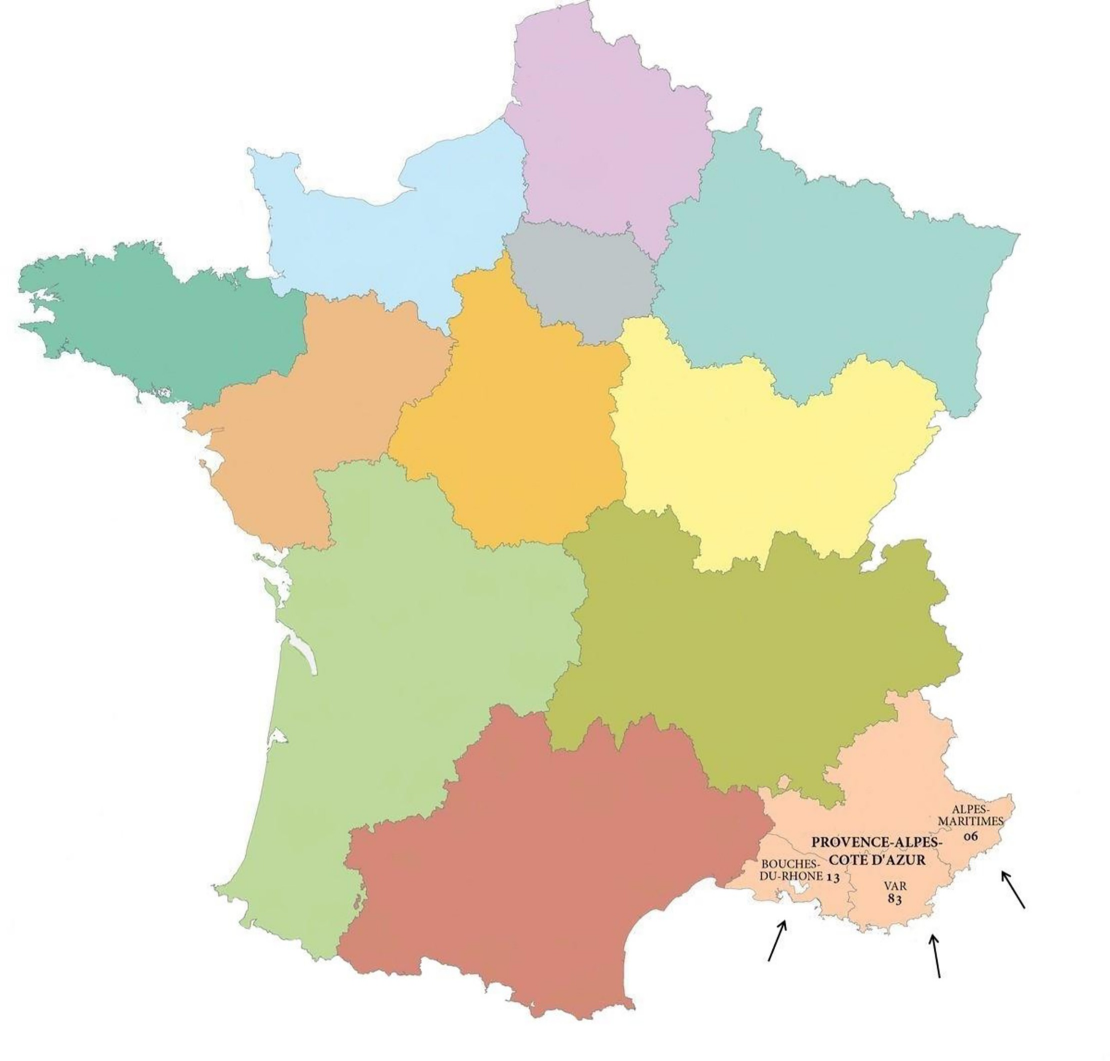
Three sub-regions in South-Eastern France involved in the study. Figure free of copyright reprinted from https://www.pacha-cartographe.fr/fonds-de-carte/. Figure is similar but not identical to the original image and is therefore for illustrative purposes only.

We included three out of the five French sub-regions constituting the South of France region Provence Alpes Côte d’Azur (PACA, Fig 1) which represent more than 4/5 of the PACA population (2,15 million in 2012 [11]). All centres referred on the official list of the Regional Agency of Health (ARS) of the PACA Region were contacted [12]. The total number of subjects attending these centres was approximately 6160 [12]. We contacted the 228 eligible centres by sending a letter explaining the study and asking for their agreement to participate in the study.

### Sample selection and inclusion of the study population

The investigators were four trained neuropsychologists specialised in schizophrenic disorders in children. Upon agreement of the eligible structures, they visited the centres in order to propose the study to all children and adolescents fulfilling inclusion criteria based on their medical records. Inclusion criteria were: age between 7 and 17 years; French speaking; IQ > 35 (WISC III/IV [13, 14]) or diagnosis of mild (F70) or moderate (F71) intellectual disability according to CIM-10 [1].

After obtaining written informed consent from the children and their parents, inclusion visits were scheduled. All subjects were included between February 2012 and May 2013.

### Data collection

We recorded socio-demographic and neonatal data, ICD diagnoses, intellectual abilities, IQ, therapeutic care, rehabilitation, socio-educational activities and pharmacological treatment, interviewed parents and reviewed the child’s medical record.

### Psychiatric diagnosis of EOS

Assessment of categorical DSM-IV-TR diagnosis of schizophrenia was undertaken using the psychosis section of Kiddie-SADS-PL [4]. Kiddie-SADS-PL is a semi-structured diagnostic interview which assesses current and past episodes of psychopathology in children and adolescents according to DSM IV-TR criteria. It consists of a first part which detects psychiatric disorders (one section per disorder) and additional sections that confirm the diagnosis. This instrument is constituted by two interviews, firstly, the parent(s) or the care-giver, and secondly, the child or adolescent; followed by a synthetic evaluation including all sources of information. When no symptoms are found, the administration of the first part takes 20 minutes. The length of the appointment increases as the number of diagnosed disorders increases, and can last up to two hours with the child and then with the adult.

### Statistical analysis

Qualitative variables are presented as percentages with 95% confidence intervals using binomial approximation of the normal distribution. Quantitative variables are presented as mean and standard deviation or median. The McNemar's Chi-squared test with continuity correction was used to compare the proportion of EOS diagnosis between the ICD and the Kiddie-SADS-PL methods. The analysis was performed using SAS software (Version 9.3 package, SAS Institute, Cary, North Carolina, USA).

## Results

### Participation of centres and inclusion of subjects

**Fig 2:**
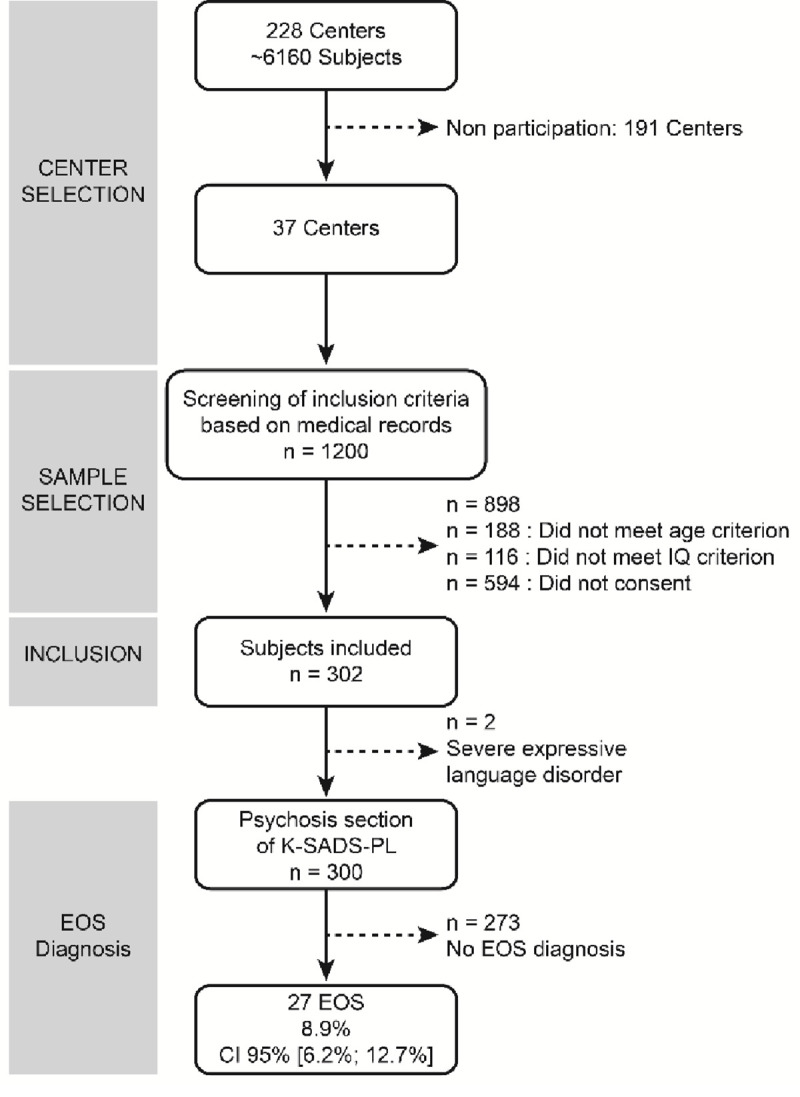
Study flow chart.

Of the 228 qualifying structures, 37 (16.2%) accepted to participate in the study. Of the total number of 1200 subjects followed up in the 37 participating centres, 896 met inclusion criteria. 302 accepted inclusion in the study (33.7%). The flow chart of the study is presented in Fig 2.

### Analyses of the total sample

#### Socio demographic characteristics (Tables 1 and 2)

Of the 302 children included, 228 (75.5%) were followed up in MSS, and 74 (24.5%) in PHS. The mean age was 11.90 years (SD = 2.8) with a sex ratio male/female of 2.8 (222 males, 80 females). Of the 302 children, 157 (52%) lived in a nuclear family setting, 78 (25.8%) in a single parent family, 49 (16.2%) in a recomposed family, and 15 (5%) were foster kids.

**Table 1:** Socio-demographic, cognitive, clinical and therapeutic description of the total sample.

		N = 302	%
**TYPE OF CENTRES**			
Psychiatric Health Sector (DH, CATTP)		74	24.5
DH		66	21.8
CATTP		8	2.6
Medico-Social Sector (IME, ITEP, SESSAD)		228	75.5
IME		108	35.8
Inpatient		48	
Outpatient		60	
ITEP		66	21.8
SESSAD		54	17.9
**SEX**			
Female		80	26.5
Male		222	73.5
**HOUSEHOLD COMPOSITION**			
Nuclear family		157	52.0
Single-parent		78	25.8
Recomposed family		49	16.2
Placement–Host family		10	3.3
Placement–Foster Home		5	1.7
Other		3	1.0
**INTELLECTUAL ABILITY**			
No Mental Retardation (IQ ≥ 70)		132	43.7
Mild Mental Retardation (50 > IQ > 69)		75	24.8
Moderate Mental Retardation (35 > IQ > 49)		76	25.2
Global IQ lacking/heterogeneity		19	6.3
**ICD-10 DIAGNOSIS in the children's file**	**ICD-10 Code**	**N = 302**	**%**
**Disorders of psychological development**	**(F80-F89)**	**105**	**32.9**
Pervasive Development Disorder	F84	67	22.2
Specific Developmental Disorder of Scholastic Skills	F81	44	14.6
**Mental retardation**	**(F70-F79)**	**69**	**21.6**
Unspecified Mental Retardation	F79	37	12.2
Moderate Mental Retardation	F71	21	6.9
**Behavioural and emotional disorders with onset usually occurring**	**(F90-F98)**	**57**	**17.9**
** in childhood and adolescence**			
Conduct Disorder	F91	44	14.6
**Disorders of adult personality and behaviour**	**(F60-F69)**	**32**	**10.0**
Specific Personality Disorders	F60	29	9.6
**Schizophrenia, schizotypal and delusional disorders**	**(F20-F29)**	**15**	**4.3**
Schizophrenia	F20	7	2.3
**Neurotic, stress related and somatoform disorders**	**(F40-F48)**	**11**	**3.4**
**Mood [affective] disorders**	**(F30-F39)**	**9**	**2.8**
**Diseases of the nervous system**	**(G00-G99)**	**6**	**1.9**
**Congenital malformations and chromosomal abnormalities**	**(Q00-Q99)**	**4**	**1.6**
THERAPIES, REHABILITATIONS AND SOCIO-EDUCATIONAL ACTIVITIES*			
**Individual Psychotherapy**		182	60
**Therapy (group or individual)**		51	16.9
Music therapy		3	
Art therapy		3	
Psycho-education		3	
Cognitive remediation		7	
Social abilities		7	
Other		29	
**Rehabilitations**		253	83.8
Speech therapy		144	47.7
Psychomotor therapy		95	59
Physical therapy		10	3.3
Occupational therapy			1.3
**Socio-Educational Activities**		178	59

DH: Day Hospitals

CATTP: Part-Time Therapy Centers

IME: Medico-Educational Institutes

ITEP: Educational and Therapeutic pedagogical Institutes

SESSAD: Specialized Home Educational and Health Care Services

IQ: Intellectual Quotient

* Possibility to cumulate several therapies and /or rehabilitations.

**Table 2:** Mean age and mean FSIQ of the total sample and mean FSIQ by sex.

		N	Mean	SD	Median	Min	Max
**Mean Age (ys)**	Total sample	302	**11.90**	2.85	12.00	7.00	18.00
Mean FSIQ*: WISC III, IV or IV abridged	Girls	57	**60.26**	18.14	55.00	38.00	110.00
	Boys	157	**68.43**	20.79	68.00	36.00	137.00
	Total	214	**66.25**	20.40	64.00	36.00	137.00

*FSIQ: Full Scale Intellectual Quotient.

#### Pregnancy and birth

Concerning pregnancy, among the 278 mothers who answered the question regarding medication or toxin intake during pregnancy, 97 (34.9%) declared having taken tobacco (69, 24.8%); alcohol (7, 2.5%), illicit drugs (5, 1.8%) or psychotropics (5, 1.8%).

Birth parameters were within the normal range. The average pregnancy term was 37.8 weeks (SD = 2.7), birth weight was 3.09 kg (SD = 6.1), birth size was 49 cm (SD = 3), and the average head circumference was 34 cm (SD = 2.2).

#### Rate of EOS in the sample

After inclusion, two children were excluded from the study because of severe language disorders not permitting assessment by Kiddie-SADS-PL.

300 participants constituted the final sample. 27 out of 300 had a diagnosis of EOS according to the Kiddie-SADS-PL psychosis section criteria, corresponding to 8.9% of the sample (CI 95%: [6.2%; 12.7%]).

#### Intellectual ability

For the majority of participants, 214/302 (70.9%), an encrypted Full Scale Intellectual Quotient (WISC III/IV [13, 14]) was recorded. For 69/302 children (22.8%), intellectual disability was recorded based on ICD-10 classification [1]. For the remaining 19/302 (6.3%) children, only some WISC subtests were available, but no Full Scale Intellectual Quotient (FSIQ). In total, 151/302 (50.0%) had intellectual disability (details in Table 1) and the average of the FSIQ was 66.3 (SD = 20.4) (Table 2). The average FSIQ among males was slightly higher than among females (p = 0.01).

#### ICD-10 diagnosis (Table 1)

Main reported ICD-10 psychiatric diagnoses notified in the medical records were: F84-Pervasive Developmental Disorders (n = 67, 22.2%); F71/F79-Mental Retardation (n = 69, 21.9%); F91-Conduct Disorder (n = 44, 14.6%), F81-Specific Developmental Disorders of scholastic skills (n = 44, 14.6%); F60-Specific Personality Disorders (n = 29, 9.6%); schizophrenia, schizotypal and delusional disorders (n = 9, 4.3%) and F20-Schizophrenia (n = 7, 2.3%).

For 214 patients (70.9%), at least one mental disease has been referenced, for 69 (22.8%) at least two, and for 30 (9.9%) at least three. 88 individuals (29.1%) had no encrypted diagnosis other than intellectual deficiency noted in their medical record.

Indeed, a significant difference (X^2^ = 16.409, df = 1, p-value < 10e-5) was found between EOS rate notified in the records (2.3%, ICD-10 diagnosis [1]) versus EOS rate diagnosed using standardized assessment (8.9%, Kiddie-SADS-PL [4]).

#### Therapeutic care, rehabilitation, socio-educational activities and pharmacological treatment (Tables 1 and 3)

182 of 302 participants (60%) had individual psychotherapy, 144 (47.7%) speech therapy, and 109 (31.5%) psychomotor, educational or physical therapy. At inclusion, 112 (37%) participants were treated with at least one medical drug, 42/112 (37.5%) took at least two, and 11/112 (9.8%) three or more drugs.

**Table 3:** Pharmacological treatment of the total sample.

Medicinal drugs	N = 112 patients	%
Allergology	5	3.8
Anti-inflammatory	5	3.8
Antibiotic	1	0.8
Cardiology	1	0.8
Endocrinology	4	3.1
Gastroenterology	2	1.5
Neurology	12	9.2
Pneumology	6	4.6
Psychiatry	90	69.2
Homeopathy	1	0.8
Metabolism-Nutrition	3	2.3
**Psychotropic drugs**		
Antipsychotics	68	53.1
Psychostimulants	15	11.7
Antiepileptics	10	7.8
Anxiolytics	12	9.4
Mood regulators	9	7.0
Antidepressants	6	4.7
Hypnotics	6	4.7
Antiparkinsonians	2	1.6

### Analyses of EOS sub-sample

#### Socio-demographic characteristics (Tables 2 and 4) and psychiatric family history

The mean age of the EOS subgroup was 12.4 years (SD = 3.25) with 14 children being younger than 13 years. The majority of children with EOS were boys (16/27, 59.3%).

**Table 4:** Socio-demographic, cognitive, clinical and therapeutic description of the EOS sub-sample.

		N = 27	%
**TYPE OF CENTRES**			
Psychiatric Health Sector (DH, CATTP)		**14**	**51.8**
DH		13	48.1
CATTP		1	3.7
Medico-Social Sector (IME, ITEP, SESSAD)		**13**	**48.1**
IME		6	22.2
Inpatient			
Outpatient			
ITEP		5	18.5
SESSAD		2	7.4
**SEX**			
Female		11	40.7
Male		16	59.3
**HOUSEHOLD COMPOSITION**		** **	** **
Nuclear family		14	51.8
Single-parent		6	22.2
Recomposed family		2	7.4
Placement–Host family		2	7.4
Placement–Foster Home		2	7.4
Other		1	3.7
**INTELLECTUAL ABILITY**			
No Mental Retardation (IQ ≥ 70)		17	63.0
Mild Mental Retardation (50 > IQ > 69)		4	14.8
Moderate Mental Retardation (35 > IQ > 49)		4	14.8
Global IQ lacking/heterogeneity		2	7.4
** ICD-10 DIAGNOSIS in the children's file**	**ICD-10 Code**	**N = 27**	**%**
**Disorders of psychological development**	**(F80-F89)**	16	59.2
Pervasive Development Disorder	F84	13	48.1
Specific Developmental Disorder of Scholastic Skills	F81	1	3.7
**Mental retardation**	**(F70-F79)**	1	3.7
Unspecified Mental Retardation	F79	1	3.1
Moderate Mental Retardation	F71	0	0
**Behavioural and emotional disorders with onset usually occurring**	**(F90-F98)**		
** in childhood and adolescence**		5	18.5
Conduct Disorder	F91	4	14.8
**Disorders of adult personality and behaviour**	**(F60-F69)**	4	14.8
Specific Personality Disorders	F60	3	11.1
**Schizophrenia, schizotypal and delusional disorders**	**(F20-F29)**	9	33.3
Schizophrenia	F20	6	22.2
**Neurotic, stress related and somatoform disorders**	**(F40-F48)**	3	11.1
**Mood [affective] disorders**	**(F30-F39)**	3	11.1
**Diseases of the nervous system**	**(G00-G99)**	0	0
**Congenital malformations and chromosomal abnormalities**	**(Q00-Q99)**	0	0
THERAPIES, REHABILITATIONS AND SOCIO-EDUCATIONAL ACTIVITIES*	** **	** **	
**Individual Psychotherapy**		20	74
**Therapy (group or individual)**		4	14.8
Music therapy		0	0
Art therapy		1	3.7
Psycho-education		0	0
Cognitive remediation		0	0
Social abilities		0	0
Other		3	11.1
**Rehabilitations**		8	29.6
Speech therapy		5	18.5
Psychomotor therapy		3	11.1
**Socio-Educational Activities**		8	29.6

DH: Day Hospitals

CATTP: Part-Time Therapy Centers

IME: Medico-Educational Institutes

ITEP: Educational and Therapeutic pedagogical Institutes

SESSAD: Specialized Home Educational and Health Care Services

IQ: Intellectual Quotient

* Possibility to cumulate several therapies and /or rehabilitations.

Fourteen out of 27 (51.8%) participants lived in a nuclear family, 6/27 (22.2%) in single-parent family, 3/27 (11.1%) in recomposed family, 2/27 (7.4%) in host family and 2/27 (7.4%) in foster care.

Concerning psychiatric family history of the 27 EOS patients, 13 (48.1%) had at least one first or second degree family member with a psychiatric history (schizophrenia, bipolar disorder, depression, autism spectrum disorder or suicide).

#### Neonatal data

Concerning the pregnancy history, 12 of 27 mothers (44.4%) admitted medication or illicit drugs during pregnancy. Three mothers (10.3%) also mentioned traumatic events that occurred during pregnancy. Concerning birth parameters, the average pregnancy term was 38 (SD = 2.4) weeks. The average birth weight was 3.29 kg (SD = 5.9), birth size 49.7 cm (SD = 3.7), and head circumference 34.1 cm (SD = 2.2).

#### Intellectual ability and ICD-10 diagnosis of EOS sample upon inclusion

The majority of the 27 children with EOS had no intellectual disability (n = 19, 70.4%), and 4 (14.8%) presented mild to moderate intellectual disability. The mean of the Full Scale Intellectual Quotient (FSIQ) was 72.5 (SD = 21.4). Four patients (14.8%) did not have any additional psychiatric diagnosis in their medical record. ICD-10 psychiatric diagnoses at inclusion are presented in Table 4.

#### Therapeutic care, rehabilitation, socio-educational activities (Table 4) and pharmacological treatment

Twenty of the 27 EOS patients (74%) followed an individual psychotherapy, 5 (18.5%) received speech therapy, 3 (11.1%) had psychomotor re-education and 8 (29.6%) participated in socio-educational activities.

Concerning the use of psychiatric medication, 14 children had received psychiatric treatment before inclusion, with one child having received several types of drugs. Before inclusion, antipsychotic drugs represented 51.5%, antiepileptics 15.1%, psychostimulant drugs 12.1%, and benzodiazepines 6% of psychotropic prescriptions. Antiepileptics were used to treat epilepsy (n = 2) or as a mood regulator (n = 6); and benzodiazepines as anxiolytic drugs. Upon inclusion, 21 of the 27 EOS patients (77.8%) took at least one psychotropic drugs with 20 patients (74%) treated with at least one antipsychotic and one patient with psychostimulant. Seven patients were treated with at least two psychotropics, and 6 had no medication at all.

## Discussion

The originality of this study is the use of standardized diagnostic assessment based on DSM IV-TR [2] in order to diagnose EOS in a large clinical sample of children and adolescents followed up in specific care structures (either in PHS or in MSS in France), half of them presenting mild to moderate intellectual disability.

### EOS diagnosis

The rate of 8.9% (CI 95%: [6.2%; 12.7%]) EOS diagnosis in this sample of children and adolescents aged 7–18 years is higher compared to 1–2% in children and 5% in adolescents of the psychiatric populations described by Remschmidt and Theisen [15]. It is much higher than the 0.2% found in a retrospective cohort study of a primary care sample using the South Carolina Medicaid Claims database [16].

The proportion of EOS diagnosis after assessment with Kiddie-SADS-PL [4] was significantly higher than in the children's medical record (8.9% vs 2.3%). The fact that diagnoses were made by different evaluators, at different ages of the patient and in different context might explain part of this discrepancy. The difference of clinical classification systems should also be considered, even if there are few in ICD- 10 [1] and DSM IV-TR [2] criteria for schizophrenia diagnosis. Nevertheless, the use of a standardized clinical assessment tool (Kiddie-SADS-PL [4]) by trained professionals specialized in childhood schizophrenia probably explains the under-diagnosis of COS in this specific paediatric population diagnosed with developmental disorders before this type of specific evaluation. Standard assessment has generally not been used in current clinical practice in medico-social or mental health structures at the time of the study. In fact, diagnosis of schizophrenia in children and adolescent is often very challenging, in particular because of its comorbidity with intellectual disability and / or ASD [7, 17, 18]. The difficulty in diagnosing EOS is also related to its overlap with developmental and other child psychiatric disorders. Other factors that need to be considered are the rarity of EOS, its similarity to mood disorders, and the presence of hallucinations in children with disorders other than schizophrenia [19]. In addition, resemblance between the course of thought disorders and infantile thoughts, as well as thought disorders due to developmental delay might also explain the risk of diagnosis error and underdiagnosis of COS.

### ICD-10 diagnosis

ICD-10 diagnosis notified in the children’s medical records was present in the vast majority of cases, but for almost 30% we did not find any clear information concerning the absence of psychiatric disorder. This could be due to cases of intellectual disability without associated psychiatric disease or an omission in the record. The most frequent diagnosis was Pervasive Development Disorders (PDD). Our results are difficult to compare because there are no recent French epidemiologic studies and existing studies do not necessarily use ICD-10 diagnosis as reference. Indeed, two studies conducted in two French sub regions in 2009 and 2012 among MSS, using CFTMEA (French classification of psychiatric disease of child and adolescent) as reference found 10–16% of autism and other PDDs, 12–13% of infantile psychosis and 22–26% of other psychiatric diseases [20, 21]. Considering that autism and infantile psychosis of the CFTMEA cover the ICD-10 chapter of PDD (F.84), the rates are 22.7% and 28% respectively, and therefore similar to the PDD rate in our sample of 22.2%.

Concerning ICD-diagnosis recorded in the medical files of the 27 children with EOS, the most common diagnosis was PDD. The result is in line with clinical overlap between ASD and EOS [22–24] and several studies describing the comorbidities associated with EOS [7, 25].

### Age

The mean age of our sample (11.90±2.8 years) was one year less than the mean age of the population in centres for disabled children and adolescents in France [11, 26]. In France, nearly half of institutionalised disabled children and adolescents are 15 to 19 years old, and 40% are 10 to 14 years old [26]. Admission to medico-social structures is rare for young children, representing less than 9% before age 10 [26]. In our sample, concerning the paediatric population between 7 and 17 years, children coming from the PHS were younger (10.6, years SD = 2.8) than those of the medico-social MSS (12.3 years, SD = 2.73).

The mean age of the children with EOS was under 13 years, and at least 50% of them presented COS.

### Sex ratio

The sex ratio sample was in favour of boys (2.8:1), accordingly to what was found in the MSS and PHS populations [11]. Along these lines, Ravaud and Ville (2003) suggested that the population of institutions for disabled children is predominantly male [27]. Lai et al. (2012 [28]) compared the prevalence of intellectual disability in children by gender in Taiwan using data from 2004 to 2010. Each year, boy cases exceeded girl cases, and the male-to-female ratio generally decreased with age [28].

The sex ratio in the EOS sample was 1.4 males for 1 female. This result is in line with those mentioned by McGrath et al., 2008, who found the same sex ratio for lifetime incidence of schizophrenia [29]. On the other hand, Stentebjerg-Olensen and colleagues described in their systematic review a preponderance of males (ratio 1.6:1) which is slightly higher than in our sample [25].

### Pregnancy and birth

Our sample reported more tobacco consumption and less alcohol use during pregnancy than the rates found in the national perinatal survey conducted in the general French population [26]. The value of the face-to-face declaration of alcohol consumption is questionable even though many prevention campaigns on the dangers of alcohol during pregnancy have been carried out in France. Birth parameters are within the standard of the French population.

### Household composition

In regards to the household breakdown, nuclear families are dominant, followed by single parents then recomposed families, as in the French population at the same period [20]. In our sample, the rate of placed children is 20 times higher than in the general population in France [27, 30]. The household composition of EOS was not different from that of the global sample.

### Cognitive assessment

The average IQ of the total sample lies in the mild intellectual disability range, which is not surprising as 35.8% of children in this sample are from IME, institutions for children with intellectual disability. The IQ of boys in our sample is higher than the IQ of girls, which can be partly explained by the fact that 23% of children are from ITEP, institutions integrating mainly boys with challenging behaviours and without intellectual disabilities.

Cognitively, the EOS sample was on average at the limit of impairment (Mean FSIQ: 72.5, SD = 21.4). This result is in concordance with most studies which found a mean FSIQ in children with EOS being between 1–2 standard deviations below the general population norm [22, 31, 32].

### Treatment

The majority of the children received reeducation and a large part followed individual psychotherapy. However, the type of psychotherapy was not identified. Almost 2/3 of children attended social and educational activities. Indeed, they are the main centres of MSS expertise. Also, there is some evidence supporting the effectiveness of psychological interventions in Early Onset Psychosis [33]. Very few children received cognitive remediation or workshops of social abilities. More than a third of children received medical drugs, specifically psychotropic drugs over half of which were antipsychotics. Nevertheless, about a quarter of children with EOS diagnoses were not treated with antipsychotics, the only available evidence-based treatment [34–36].

### Limitations

We encountered a high rate of non-participation of centres (83.7%). Several reasons should be discussed. Firstly, some French psychiatrists might be opposed to standardized diagnosis criteria based on atheorical classifications. Secondly, some physicians might have been worried by the families’ reaction to possible EOS diagnosis revealed by the study. Thirdly, some directors of MSS might have been concerned with regards to the staff’s reactions upon a diagnosis of schizophrenia.

We found a high rate of nonparticipation of families in the study (66.3%). In fact, many parents were themselves in precarious social situations and reluctant to devote time to this study which might explain part of this result. COS is a very challenging disease for diagnosis as well as follow-up with a high impact on patients but also on families. Compared to the literature, Lépine et al. (2000), also found a similar high nonparticipation rate (64%) in a study of the prevalence of psychiatric disorders and comorbidities study in the French general population, the ESEMED study [37]. Morton and colleagues (2005), found the reporting of participation varied significantly by type of epidemiologic studies and the participation information was not provided in any of the retrospective cohort studies [38]. Galea and Tracy (2007) observed that the participation rates for epidemiologic studies had steady declined over the past 30 years [39]. Thus, they showed that in ten years the participation rate reported in the epidemiological studies of prevalence of psychiatric disorders the National Comorbidity Survey (NCS) and NCS-replication decreased from 82.4% to 70.9%.

Because of the low participation rate of centres and families, the sample might not be sufficiently representative of the study population and the results can therefore not be extrapolated. However, study results underline the importance of improving the diagnosis of EOS in the pediatric population with early developmental disorders in France. Our study demonstrates that the assessment of EOS diagnosis by standardized diagnostic tools involving the child, parents as well as the support team, is feasible in the pediatric population with neurodevelopmental disorders and intellectual deficiency. In order to adapt therapeutics and care of EOS children, follow-up assessments with standardized instruments covering clinical symptoms and functioning should be implemented in PHS and MSS centers. A correct and early diagnosis of COS using standardized assessment tools such as K-SADS-PL [4] seems also very important in order to allow early prescription of antipsychotic treatments, the only available evidence-based treatment in this indication [34–36]. Nevertheless, pharmacological treatment should not be a single treatment approach for these very complex patients and families.

## Conclusion

The results of this study highlight the importance of using a standardized diagnostic tool for the diagnosis of schizophrenia in the paediatric population. In fact, EOS might be underdiagnosed in children and adolescents with neurodevelopmental disorders and subnormal cognitive abilities. Indeed, diagnostic assessment of schizophrenia in children is very challenging and should involve children, parents as well as support teams. Nevertheless, the fact that this disorder is often unrecognised seems to lead to suboptimal interventions which are likely to have repercussions on long term outcomes. We should therefore ensure that diagnosis of COS and its comorbidities are based on robust standardized assessment in order to enable the early use of validated treatments such as antipsychotics and to adjust therapies, thus improving short, medium and long term prognosis and outcome for children with COS.
